# Paternal Age and Transgenerational Telomere Length Maintenance: A Simulation Model

**DOI:** 10.1038/s41598-018-36923-x

**Published:** 2019-01-10

**Authors:** K. Horvath, D. Eisenberg, R. Stone, J. Anderson, J. Kark, A. Aviv

**Affiliations:** 1 Center of Human Development and Aging, Rutgers, The State University of New Jersey, New Jersey Medical School, Newark, New Jersey United States of America; 2 Department of Anthropology, and Center for Studies in Demography and Ecology, University of Washington, Seattle, Washington United States of America; 3 University of Washington, School of Aquatic and Fishery Sciences, Seattle, Washington United States of America; 4 Hebrew University-Hadassah School of Public Health and Community Medicine, Jerusalem, Israel

## Abstract

Telomere length (TL) in offspring is positively correlated with paternal age at the time of the offspring conception. The paternal-age-at-conception (PAC) effect on TL is puzzling, and its biological implication at the population level is unknown. Using a probabilistic model of transgenerational TL and population dynamics, we simulated the effect of PAC on TL in individuals over the course of 1,000 years. Findings suggest a key role for an isometric PAC midpoint (PACmp) in modulating TL across generations, such that offspring conceived by males younger than the isometric PACmp have comparatively short telomeres, while offspring conceived by males older than the isometric PACmp have comparatively long telomeres. We further show that when cancer incidence escalates, the average PAC drops below the isometric PACmp and transgenerational adaptation to cancer ensues through TL shortening. We propose that PAC serves to maintain an optimal TL across generations.

## Introduction

Large, long-living mammals typically display short telomeres and repressed telomerase in somatic tissues^1–4^. Short telomeres can limit proliferative activity, diminishing the burden of cancer driver mutations and thereby attenuating cancer risk. However, short telomeres (and reduced telomerase activity) also increase predisposition to degenerative diseases, particularly in older animals. Converging evidence suggests that this cancer-degenerative disease trade-off operates not only across mammals but also within *Homo sapiens*^1^, as constitutively long telomeres^5–8^ and alleles that associate with long telomeres^9–13^ increase the risk of some cancers, while constitutively short telomeres^14–16^ and alleles that associate with short telomeres^13,17,18^ increase the risk of adult-onset cardiovascular disease (CVD), the main degenerative disease afflicting contemporary humans^19^. At the population level, across generations and in the face of changing environments (e.g., due to human migratory tendencies), this telomere length (TL)-mediated cancer-degenerative disease trade-off might require meticulous maintenance.

Most of the empirical data on human TL is based on leukocyte TL (LTL), which reflects TL in other somatic cells^20^. LTL is a highly heritable^21–23^ complex genetic trait that exhibits wide inter-individual variation from birth onwards^24,25^. In previous work, we proposed that polygenetic adaptation is one of the mechanisms that maintain the TL-mediated cancer-degenerative trade-off^26^. Here we propose, and illustrate by simulations, that the effect of paternal age at conception (PAC) on the offspring’s TL might be another mechanism that contributes to the maintenance of this trade-off.

The PAC effect, expressed as a longer LTL in offspring conceived by older men^23,27–31^, apparently stems from longer telomeres in sperm of older men^31,32^. This contrasts with the general phenomenon of telomere shortening with age in somatic tissues. Telomerase, the reverse transcriptase that elongates telomeres^33^, and sperm selection are potential explanations for the longer TL in sperm of older men. Telomerase, which is largely silent in somatic tissues, is active in the testes and presumably in the male germ stem cells^34^. Analysis of the PAC effect in twins also suggests the possibility of an age-dependent selection of sperm with longer telomeres^29^. The longer telomeres in the male germline are apparently inherited by the offspring according to Mendelian principles^35,36^.

While the effects of PAC on offspring’s TL across one^24,27–29,31^ and two^30^ generations have been studied, little is known about how PAC impacts TL dynamics across multiple generations. To gain insight into potential implications of the PAC effect for future TL trajectories, we incorporate the PAC effect into a simulation model of transgenerational TL that integrates the effects of heritability, PAC and cancer on TL.

## Results

### Model basic parameters

The model comprises a founding population (n = 1,000), male-female mating, fertility based on female and male ages, birth and death (Materials and Methods; Supplementary Figs S1 and S2). As TL is highly heritable^21–24^, and as studies showed that maternal inheritance is stronger than paternal inheritance^23,24^, the model applies a set of assumptions, including: (a) a mode of TL inheritance, whereby each parent’s genetic makeup contributes directly to the offspring’s TL using an inheritance factor that weights the maternal and paternal contribution to the offspring’s TL based on empirical observations (Materials and Methods)^21–24^; (b) the PAC effect of 15 base pairs (bp) per each additional year of paternal age^24,27–29,31^; (c) age-dependent TL shortening^16,20,21,28,31,32,37^; and (d) a stochastic factor based on a standard deviation of TL observed across newborns^24,25^ in which the TL-outcome is normally distributed (mean = 0 bp; SD = 700 bp).

We used Monte Carlo simulation to drive the model and track TL of the population with/without the PAC effect and with/without TL-dependent cancer. Vital parameters, e.g., mortality, fertility, etc., were derived from CDC data (Supplementary Table S1)^38,39^. To make the simulations computationally tractable in the face of exponential growth, we restricted the transgenerational timespan to 1,000 years and imposed a constraint on the maximum number of lifetime births per individual (Materials and Methods).

The initial series of simulations covered a range of birth TLs (7,000–11,000 bp) in the founding population, but key simulations focused on birth TL of 9,500 bp, the approximate birth TL empirically observed in humans^2,24,25^. A key parameter in the simulations was the ‘telomeric brink’ (TB), i.e., TL = 5,000 bp, which denotes a 50% probability of imminent death because of compromised ability to maintain homeostasis through cell replication (Supplementary Fig. S3)^40,41^. The TB (Materials and Methods) is based on the average TL observed in dyskeratosis congenita, a monogenic disease with critically short TL due detrimental mutation in telomere maintenance genes^41^. Another key parameter was TL-associated cancer (and its consequential mortality). This parameter is based on formulation of TL-dependent cancer incidence in which the probability of acquiring cancer, presumably due to the accumulation of driver mutations, in the course of a given year is a function of an individual’s age and TL^40^.

### Simulations without cancer

**Figure 1:**
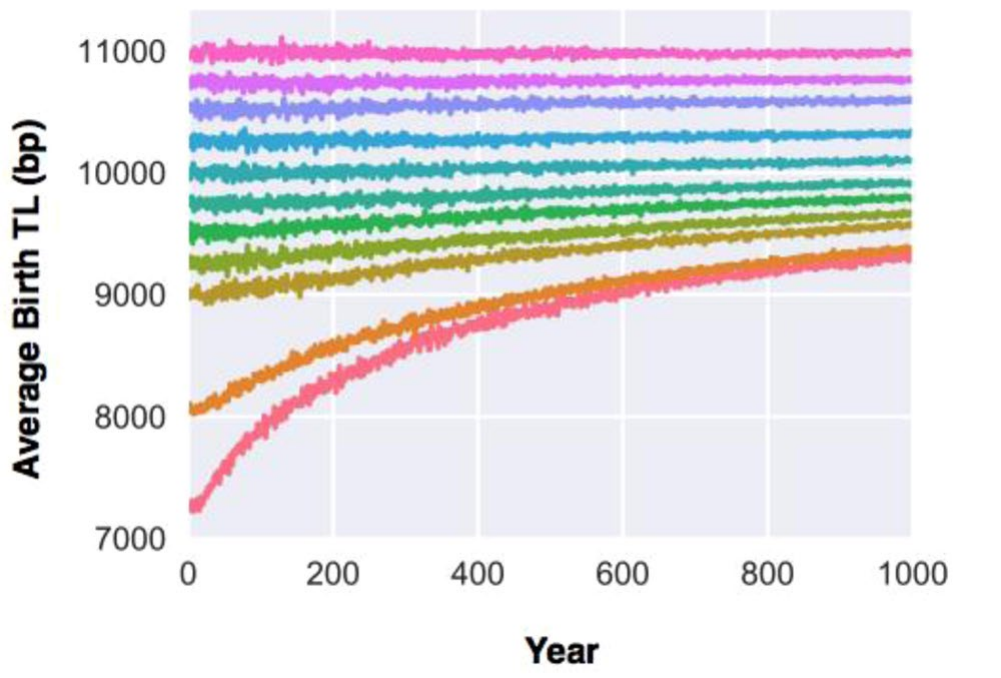
Transgenerational telomere length (TL) dynamics in the presence of the telomeric brink (TB) over the course of 1,000 years. Telomere length at birth for the founding population ranges between 7,000–11,000 bp.

Our first series of simulations focused on TL dynamics in the presence of the TB, but without cancer or the PAC effect, over the course of 1,000 years for birth TLs of 7,000–11,000 bp in the founding population. The selective pressure exerted by the TB, together with the mixing TLs through inheritance, plus the stochastic effect across the population, resulted in upward birth TL trajectories whose steepness was inversely related to mean birth TL in the founding population (Fig. 1).

**Figure 2:**
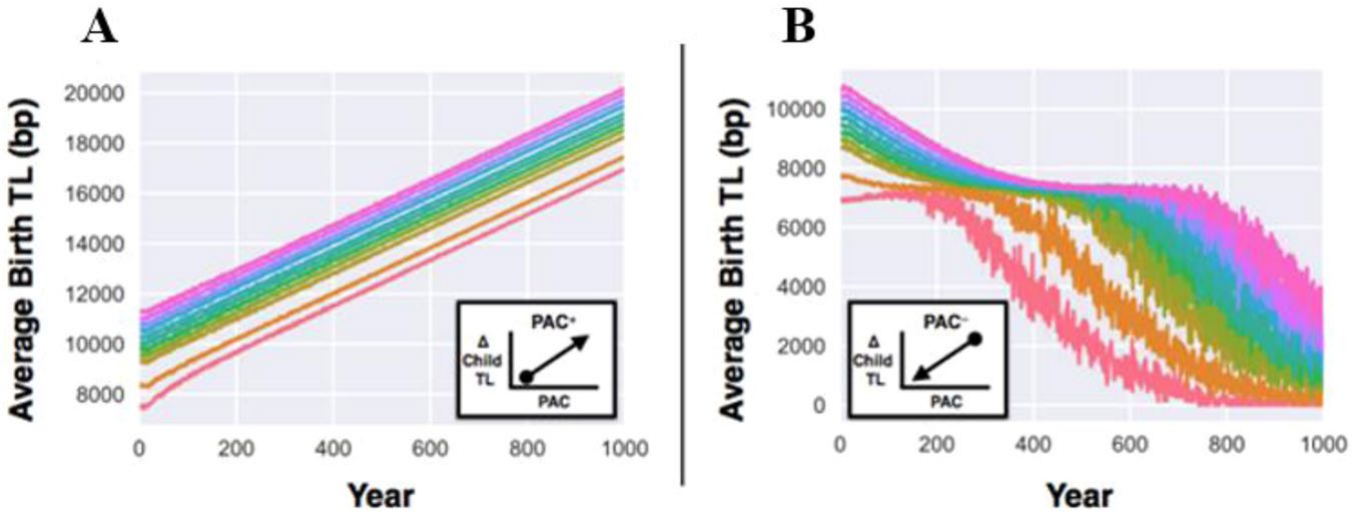
Transgenerational telomere length (TL) dynamics in the presence of the telomeric brink (TB) and the paternal age at conception (PAC) effect over the course of 1,000 years. TL at birth for the founding population ranges between 7,000–11,000 bp. For (**A**), PAC effect on the offspring’s TL is modelled as an exclusively additive process (PAC^+^), while for (**B**), the PAC effect on the offspring’s TL is modelled as an exclusively subtractive process (PAC^−^). Note that after 500 years, the fusion of colors for the downward trajectories of TL at birth >8,000 bp is due to wide TL oscillation.

We then repeated these simulations with the PAC effect functioning in two independent modes: (i) offspring conceived by older fathers having a longer TL (a PAC^+^ mode), presumably because TL is longer in sperm of older men (Fig. 2A), and (ii) offspring conceived by younger fathers having a shorter TL (a PAC^−^ mode), presumably because TL is shorter in sperm of younger men (Fig. 2B). The PAC^+^ mode further increased the upward transgenerational TL trajectories without bound, while the PAC^−^ mode caused a reversal of the TL trajectories downward to the TB. For mean TL at birth = 7,000 bp in the PAC^−^ mode, the initial transgenerational trajectory was slightly upward, then assuming a downward course with wide oscillation (i.e. year-to-year variance). For all other mean TLs at birth in the PAC^−^ mode, the trajectories first descended towards ~7,000 bp with minimal TL oscillations and then plunged downward with wide TL oscillations. These configurations reflect the independent and opposing pressures of the TB (upward) and the PAC^−^ effect (downward) on the transgenerational TL trajectories. The widening TL oscillations with the passage of time stems from the contraction of sample size as the population moves towards extinction due to critically short TL.

**Figure 3:**
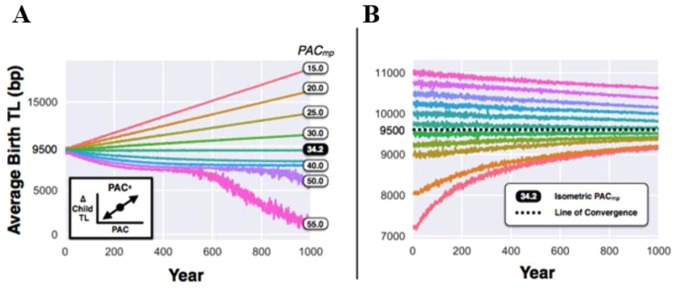
Bidirectional paternal age at conception (PAC) effect on transgenerational telomere length (TL) dynamics over the course of 1,000 years. For (**A**), simulations of the PAC effect are modelled over a range of PAC midpoints (PAC_mp_) as a mixed additive and subtractive process (PAC^±^). For a founding population with birth TL = 9,500 bp, there exists one isometric PAC_mp_ (34.2 years), such that transgenerational TL is neither increasing nor decreasing. For (**B**), when PAC_mp_ is held at isometric PAC_mp_, for founding populations with birth TLs between 7,000–11,000 bp, the transgenerational TL dynamics apparently converges towards 9,500 bp.

Clearly, whether in the positive or negative mode, a unidirectional PAC effect exerts a ‘destabilizing’ influence on the transgenerational TL trajectory. For this reason, focusing on mean birth TL of 9,500 bp, we tested a simultaneously positive and negative PAC effect, i.e., a bidirectional (PAC^±^) mode in which the relative change in the offspring TL is determined with regard to a fixed age about which the PAC effect is centered, i.e. a PAC-midpoint (PAC_mp_). In this mode, when paternal age was older than the PAC_mp_, it acted in a PAC^+^ mode, lengthening TL in the offspring by 15 bp/year for each paternal year greater than PAC_mp_; when PAC age was younger than the PAC_mp_, it acted in a PAC^−^ mode, shortening TL in the offspring by 15 bp/year for each paternal year less than PAC_mp_. Thus, when the mean PAC for a population is above the PAC_mp_, there is an overall transgenerational lengthening of population TL; in contrast, when the mean PAC is below the PAC_mp_, there is an overall transgenerational shortening of population TL (Fig. 3A). As per the PAC^−^ model (Fig. 2B), the wider TL oscillations for PAC_mp_ = 50 or 55 years reflects the contraction of sample size as the population moves towards extinction due to critically short TL.

Through simulations, we then found the PAC age for a founding population with mean birth TL = 9,500 bp, where the transgenerational TL trajectory was flat (i.e., at equilibrium). We refer to this PAC as the isometric PAC_mp_ (34.2 years; Fig. 3A). Next, we examined the effect of this specific isometric PAC_mp_ on mean birth TLs ranging between 7,000–11,000 bp in a founding population. At this PAC_mp_, the transgenerational TL trajectories converged toward 9,500 bp (Fig. 3B). These findings underscore a key outcome of the model, namely, PAC_mp_ and mean population TL are inter-connected traits. In this way, isometric PAC_mp_ drives convergence of the mean population TL toward the initial TL from which the isometric PAC_mp_ is derived.

### Simulations with cancer

To illustrate the PAC-environment interaction, our next series of simulations were based on theoretical scenarios of a changing environment wherein the cancer incidence escalates, e.g. due to an increase in the cumulative mutation load. Focusing on mean birth TL of 9,500 bp (year 0), we simulated the transgenerational TL trajectories, based on the following settings: baseline cancer (scale 1), and increased cancer incidence (scales 2 and 4) in which the cancer incidence was scaled upward 2 and 4 fold, respectively. For each cancer incidence scale, we simulated the transgenerational TL in response to increased cancer based on two sub-scenarios: (a) without the PAC effect, and (b) with the PAC effect. To this end, we allowed the model to operate without PAC for the first 500 years, and then introduced the PAC effect into the model beginning at year 500.

**Figure 4:**
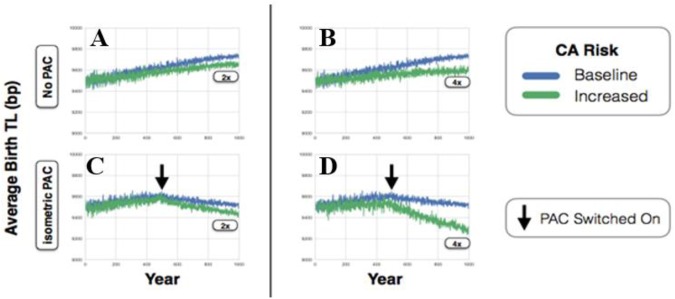
Transgenerational telomere length (TL) dynamics over the course of 1,000 years in the presence of baseline (blue) or increased (green) of cancer risk. For (**A**–**D**), starting at baseline, birth TL in the founding population is 9,500 bp. For (**A**,**B**), simulations of transgenerational TL dynamics are displayed without the paternal age at conception (PAC) effect. For (**C**,**D**), simulations of transgenerational TL dynamics are displayed with the PAC effect at the isometric PAC_mp_ (34.2 years), which is introduced at 500 years (vertical arrow). Increased cancer risks are at 2-fold (**A** and **C**) to 4-fold (**B** & **D**) scale factor.

In the absence of the PAC effect, increased cancer attenuated the upward transgenerational TL trajectories due to the upward pressure imposed by the TB (Fig. A,B). However, even in the face of a four-fold increase in cancer incidence, the transgenerational TL trajectory remained upward, suggesting that the TB continued to drive up TL and thereby increase cancer incidence. In contrast, introduction of the PAC^±^ effect (at isometric PAC_mp_) not only stabilized the transgenerational TL in the absence of cancer, but also shifted the direction of the transgenerational TL trajectory downward in the presence of cancer. This PAC effect was proportional to the magnitude of the increase in cancer incidence (Fig. 4C,D), presumably because older individuals were likely to die from the disease, which thereby decreased the mean PAC of the population.

**Figure 5:**
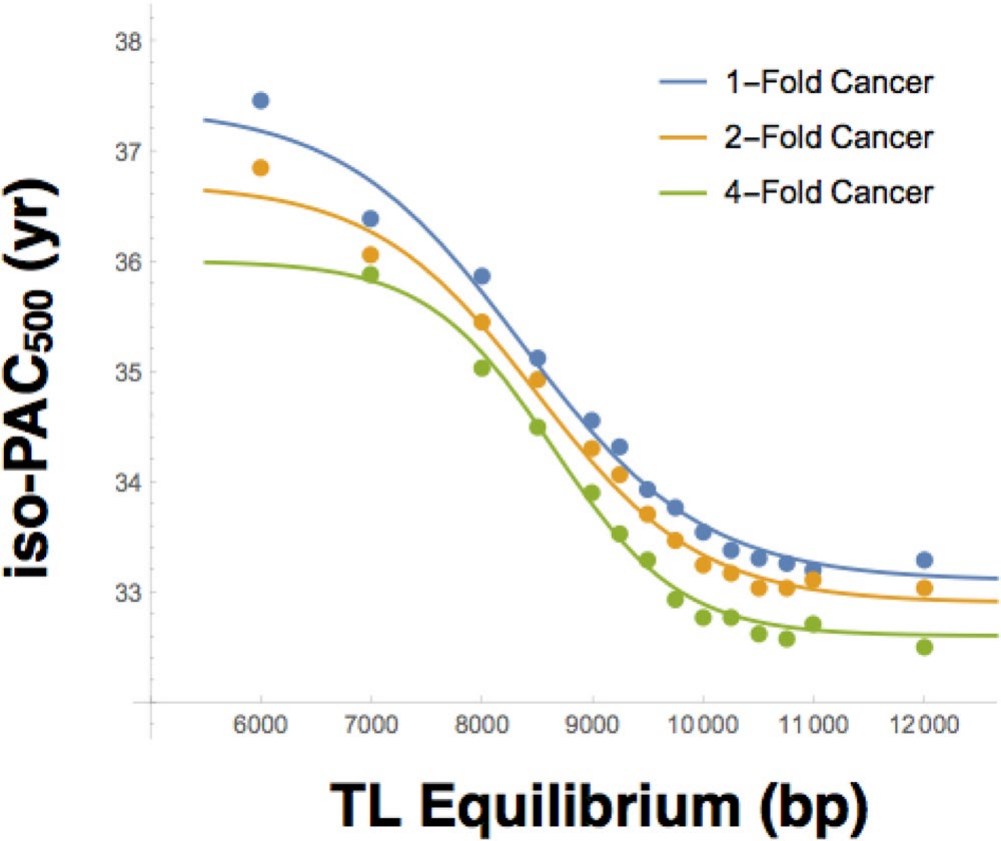
Isometric paternal age at conception midpoint (PACmp), relative to year 500 (iso-PAC500) vs. birth telomere length (TL) Equilibrium. Simulations are performed for mean birth TL between 6,000–15,000 bp in the founding population for models with 1-Fold (blue), 2-Fold (yellow), and 4-Fold (green) cancer scaling factors. iso-PAC500 is taken as the PACmp for which mean transgenerational TL is unchanged between years 500 and 1000. Results are fit to a logistic curve and plotted for TL ranging between 6,000 and 12,000 bp.

To further understand the relation between PAC and the transgenerational TL dynamics in the face of cancer, we expanded the scope of the simulations to include founding populations with mean birth TL ranging from 6,000 to 15,000 bp (year 0). For each mean birth TL, we identified a unique isometric PAC_mp_ that stabilized the transgenerational TL for the period during which the PAC effect was included in the simulation (i.e. years 500–1000), denoted as iso-PAC_500_. The relationship between each iso-PAC_500_ and this TL was then plotted for TL-dependent cancers at scales 1, 2, and 4-fold. Results were fitted to logistic curves and exhibited two key features (Fig. 5): First, a younger iso-PAC_500_ maintained a comparatively long equilibrium TL. However, the relation between the two variables was nonlinear and complex. This likely reflects the opposing selective pressures of the TB, pushing TL upward from the bottom of the TL distribution, and cancer, pushing TL downward from the top of the distribution. Second, as the cancer scale factor increased, the iso-PAC_500_ decreased. Taken together, findings suggest that the iso-PAC_500_ and equilibrium TL are related within a given environment, whereby a change in one determines a change in the other.

## Discussion

The PAC effect may apply across the primate lineage, since it is also observed in chimpanzees^42^. This begs the question why the PAC effect has evolved. Our simulations show an upward transgenerational TL trajectory when the PAC effect is expressed in the inheritance of comparatively long telomeres by offspring of older fathers, i.e., PAC^+^ (Fig. 2A), and a downward trajectory when the PAC effect is expressed in the inheritance of comparatively short telomeres in offspring of younger fathers, i.e., PAC^−^ (Fig. 2B). A bidirectional (PAC^±^) and isometric PAC_mp_ effect (Fig. 3) engender stability of TLs across generations and are thus more evolutionarily likely. The biology of the male germline affords the evolution of this effect, since testes are formed in utero and sperm production commences at puberty, forging functional sperm shortly thereafter and throughout the remaining life course^43^. Given the numerous replications of stem cells, which continue unabated in the male germline, sperm TL may be amenable to modification^29,43,44^.

Over the course of human evolution, the isometric PAC_mp_ has probably fluctuated near the mean age of male reproduction. In light of increasing mean PAC in recent decades in many western countries^45–47^, we might expect that TL has been progressively increasing across generations. However, several decades represent a tiny period in human evolution. Comprehensive historical analyses show, for instance, that in the US mean PAC declined considerably between 1876 and 1979 (from 36.9 years to 27.9 years) and only started to increase after 1979^48^. Estimates of historic and pre-historic paternal age, which are likely more evolutionarily informative, are difficult to come by. In contemporary hunter gatherer populations, mean PAC is approximately 40 years^49^, suggesting that TL may have been getting successively shorter in western countries over the past century and that the recent rise in PAC has reversed this trend.

Another major finding of our simulations is that the PAC effect and cancer can work interactively because older men are more likely to die from cancer, thereby shifting downward the PAC distribution and thus TL. It follows that the reverse might also be true, i.e., if later life survival increased, the distribution of PAC and thus TL would be shifted upward. Such an effect on TL might allow relatively rapid changes towards an optimal TL in response to environmental and demographic conditions. We note in this regard that in contemporary humans, both cancer and CVD primarily occur during the post-reproductive years. Natural selection, mediated via factors such as polygenetic adaptation^26^ and PAC, may have adjusted in relatively short time periods the optimal TL to balance cancer against degenerative diseases and lessens their impact during the reproductive years.

Since the cancer-degenerative disease trade-off is a prominent explanation for TL evolution and there are clear empirical precedents to build on, our simulation model focuses on these factors. Another key driver of the optimal TL might be exposure to infection and thus TL-dependent immune function during early life and thereafter. In present societies, drivers of the PAC effect on TL may also act through demographic pathways. For instance, the age of conception is positively correlated with social-economic status^50–52^, life expectancy^53^ and inversely with early-life poor health^54^. Thus, correlations of TL with health prior to and during reproductive ages may affect PAC. Moreover, cancer in older family members may affect the PAC of younger members since cancer impacts both family economic resources^55^ and family member health^56^. Such PAC drivers are expected to be complex but may be significant and fast acting mechanisms for population adaptation in a changing environment.

Finally, we hope that our model will be further refined and serve as a catalyst for testing through simulations the impact of diverse environmental and demographic settings on the optimal TL in the human population.

## Materials and Methods

### General approach

Our model is based on empirical observations from epidemiological studies and theoretical considerations, as described under Results. It comprises population-level processes that are incorporated into four modules (Supplementary Figs S1,S2). The first module characterizes each individual (virtual human) by sex, date of birth, date of death, birth TL, and the rate of age-dependent TL attrition; together, birth TL and TL attrition define each individual’s TL throughout his/her life course. Based on cross-sectional and longitudinal studies of age-dependent TL shortening^16,20,21,28,31,32,37^, the attrition in TL during the first two decades is taken as 1,400 bp and at 25 bp per year thereafter.

The second is a module of TL inheritance, whereby a newborn’s birth TL is specified by a combination of independent processes. One process specifies TL at birth as a weighted average of the maternal and paternal birth TLs, respectively. The weights are given by a maternal inheritance factor of 57.5%^24^. Next, to account for natural TL variance, a stochastic process is used to adjust the birth TL by a random amount taken from a normal distribution with standard deviation 700 bp^24,25^. The PAC effect is taken as 15 bp/yr^24,27–29^. For PAC^+^ models, the isometric PAC midpoint (PAC_mp)_ is given as 15 years (the minimum PAC in the fecundity module below). Thus, all fathers older than 15 years will endow their offspring with increasingly longer TLs. For PAC^−^ models, the PAC_mp_ is given as 55 years (the maximum PAC in the fecundity module). Thus, all fathers younger than 55 years will endow their offspring with shorter TLs. Intermediate models for the PAC effect are similarly parameterized by a PAC_mp_ between 15 and 55 years (Supplementary Fig. S4).

The third module, fecundity, accounts for the distribution of maternal and paternal ages. This model assumes that all living females become pregnant with a probability distribution parameterized solely by age (Supplementary Table S1). Male fertility is similarly modelled in an all-or-none fashion, such that fertile males have uniform probability of a successful mating during their reproductive years and are infertile otherwise. Due to computational limitations imposed in proportion to population size, rapid population expansion was attenuated by arbitrarily restricting each female to a maximum of 3 lifetime births to facilitate a simulated period of 1,000 years.

The fourth module, mortality, determines the individual’s death year, which may be ascribed to one of three independent processes. A baseline mortality rate is taken as the probability that an individual dies in a given year. Baseline mortality is parameterized only by age (Supplementary Table S1), and is therefore independent of TL-related causes of mortality. TL-dependent causes of death are modeled independently based on whether the effect is proportional or inversely proportional to TL (i.e. effects that are more likely to occur in individuals with relatively longer telomeres vs. effects that are more likely to arise in individuals with relatively shorter telomeres). Because TL must be always positive, the probability of death must approach 100% as TL approaches zero. Causes of death linked to short TL, therefore, fall under a TB Model, whereby TL is inversely correlated with probability of death. This is characterized as a logistic function over TL centered at 5,000 bp (Supplementary Fig. S3). Conversely, death related to long TL is determined by a Cancer Model, which is parameterized by both age and birth TL. (This model is based on equation 8 in supplement^40^. These potential causes of death are independently simulated for each year of the individual’s life and the earliest cause of death determines the age at death of the individual. Because birth TL is always positive and finite, the TB model guarantees that all individuals will eventually die.

Together, these four modules were run through Monte Carlo simulation (Supplementary Fig. S2) written in the Scala programming language, with an initial population consisting of 500 males and 500 females and allowed to proceed for a period of 1,000 cycles (years). The state of the population was tracked year-over-year in the simulator and summary statistics generated corresponding to mean newborn TL. The simulation itself was run for multiple module variants and parameterized by trial number, presence or absence of a PAC effect with corresponding PAC_mp_, TL-dependent cancer module (including a scaling factor for arbitrarility increasing cancer risk), maternal inheritance, fecundity module, mortality module, and a constant birth TL for the founding population. Each module was then run in parallel on the Rutgers University Harpertown High Performance Computing Cluster at Rutgers University, consisting of approximately 300 compute nodes each outfitted with an 8-core Xeon 2.83 GHz (Intel E5440) Processor, and 16 GB memory. Finally, the outputs from each of these simulations (50 runs per scenario) were combined for analysis.

Early simulations of the PAC effect displayed an abrupt initial decline in trangenerational TL with wide variance which stabilized over the course of several hundred cycles. This decline most likely arose from the model starting with only young individuals (since the model had to run longer for individuals to age) and only young men thus being able to reproduce initially. To overcome this artifact, we constructed a founding population with a heterogeneous age distribution as follows: 100 individuals were generated each year over the course of 100 years (total = 10,000 individuals) prior to initiating the simulation. Of the surviving population, 1,000 individuals were randomly chosen to establish the founding population. In this way, the founding population reflects the wide age distribution arising from the mortality module.

## Supplementary information

Supplementary Information

## Data Availability

The code of our model is available at http://njms.rutgers.edu/centers_institutes/humandevelopmentandaging/.

## References

[1] Stone RC (2016). Telomere Length and the Cancer-Atherosclerosis Trade-Off. PLoS genetics.

[2] Gomes NM (2011). Comparative biology of mammalian telomeres: hypotheses on ancestral states and the roles of telomeres in longevity determination. Aging cell.

[3] Seluanov A (2007). Telomerase activity coevolves with body mass not lifespan. Aging cell.

[4] Risques, R. A. & Promislow, D. E. L. All’s well that ends well: why large species have short telomeres. *Philosophical transactions of the Royal Society of London. Series B, Biological sciences* **373**, 10.1098/rstb.2016.0448 (2018). 10.1098/rstb.2016.0448 PMC5784068 29335372

[5] Anic GM (2013). Telomere length and risk of melanoma, squamous cell carcinoma, and basal cell carcinoma. Cancer epidemiology.

[6] Seow WJ (2014). Telomere length in white blood cell DNA and lung cancer: a pooled analysis of three prospective cohorts. Cancer research.

[7] Julin B (2015). Circulating leukocyte telomere length and risk of overall and aggressive prostate cancer. British journal of cancer.

[8] Zochmeister C (2018). Leukocyte telomere length throughout the continuum of colorectal carcinogenesis. Oncotarget.

[9] Walsh KM (2015). Longer genotypically-estimated leukocyte telomere length is associated with increased adult glioma risk. Oncotarget.

[10] Zhang C (2015). Genetic determinants of telomere length and risk of common cancers: a Mendelian randomization study. Human molecular genetics.

[11] Machiela MJ (2015). Genetic variants associated with longer telomere length are associated with increased lung cancer risk among never-smoking women in Asia: a report from the female lung cancer consortium in Asia. International journal of cancer.

[12] Iles, M. M. *et al*. The effect on melanoma risk of genes previously associated with telomere length. *Journal of the National Cancer Institute* **106**, 10.1093/jnci/dju267 (2014). 10.1093/jnci/dju267 PMC4196080 25231748

[13] Haycock PC (2017). Association Between Telomere Length and Risk of Cancer and Non-Neoplastic Diseases: A Mendelian Randomization Study. JAMA oncology.

[14] D’Mello MJ (2015). Association between shortened leukocyte telomere length and cardiometabolic outcomes: systematic review and meta-analysis. Circulation. Cardiovascular genetics.

[15] Haycock PC (2014). Leucocyte telomere length and risk of cardiovascular disease: systematic review and meta-analysis. BMJ (Clinical research ed.).

[16] Benetos, A. *et al*. Short Leukocyte Telomere Length Precedes Clinical Expression of Atherosclerosis: Blood-and-Muscle Model. *Circulation research*, 10.1161/circresaha.117.311751 (2017). 10.1161/CIRCRESAHA.117.311751 PMC5821479 29242238

[17] Codd V (2013). Identification of seven loci affecting mean telomere length and their association with disease. Nature genetics.

[18] Zhan Y (2017). Exploring the Causal Pathway From Telomere Length to Coronary Heart Disease: A Network Mendelian Randomization Study. Circulation research.

[19] Savage, S. A. Beginning at the ends: telomeres and human disease. *F1000Research* **7**, 10.12688/f1000research.14068.1 (2018). 10.12688/f1000research.14068.1 PMC5931273 29770205

[20] Daniali L (2013). Telomeres shorten at equivalent rates in somatic tissues of adults. Nature communications.

[21] Hjelmborg JB (2015). The heritability of leucocyte telomere length dynamics. Journal of medical genetics.

[22] Slagboom PE, Droog S, Boomsma DI (1994). Genetic determination of telomere size in humans: a twin study of three age groups. American journal of human genetics.

[23] Broer L (2013). Meta-analysis of telomere length in 19,713 subjects reveals high heritability, stronger maternal inheritance and a paternal age effect. European journal of human genetics: EJHG.

[24] Factor-Litvak, P. *et al*. Leukocyte Telomere Length in Newborns: Implications for the Role of Telomeres in Human Disease. *Pediatrics* **137**, 10.1542/peds.2015-3927 (2016). 10.1542/peds.2015-3927 PMC4811318 26969272

[25] Aubert G, Baerlocher GM, Vulto I, Poon SS, Lansdorp PM (2012). Collapse of telomere homeostasis in hematopoietic cells caused by heterozygous mutations in telomerase genes. PLoS genetics.

[26] Hansen ME (2016). Shorter telomere length in Europeans than in Africans due to polygenetic adaptation. Human molecular genetics.

[27] Unryn BM, Cook LS, Riabowol KT (2005). Paternal age is positively linked to telomere length of children. Aging cell.

[28] De Meyer T (2007). Paternal age at birth is an important determinant of offspring telomere length. Human molecular genetics.

[29] Hjelmborg JB (2015). Paternal age and telomere length in twins: the germ stem cell selection paradigm. Aging cell.

[30] Eisenberg DT, Hayes MG, Kuzawa CW (2012). Delayed paternal age of reproduction in humans is associated with longer telomeres across two generations of descendants. Proceedings of the National Academy of Sciences of the United States of America.

[31] Kimura M (2008). Offspring’s leukocyte telomere length, paternal age, and telomere elongation in sperm. PLoS genetics.

[32] Aston KI (2012). Divergence of sperm and leukocyte age-dependent telomere dynamics: implications for male-driven evolution of telomere length in humans. Molecular human reproduction.

[33] Blackburn EH, Epel ES, Lin J (2015). Human telomere biology: A contributory and interactive factor in aging, disease risks, and protection. Science (New York, N.Y.).

[34] Wright WE, Piatyszek MA, Rainey WE, Byrd W, Shay JW (1996). Telomerase activity in human germline and embryonic tissues and cells. Developmental genetics.

[35] Baird DM, Rowson J, Wynford-Thomas D, Kipling D (2003). Extensive allelic variation and ultrashort telomeres in senescent human cells. Nature genetics.

[36] Graakjaer J (2006). Allele-specific relative telomere lengths are inherited. Human genetics.

[37] Steenstrup T, Hjelmborg JV, Kark JD, Christensen K, Aviv A (2013). The telomere lengthening conundrum–artifact or biology?. Nucleic acids research.

[38] Centers for Disease Control and Prevention/National Center for Health Statistics (CDC/NCHS), National Vital Statistics System, Mortality. Retrieved from http://www.cdc.gov/nchs/data/dvs/lcwk1_2014.pdf (2014).

[39] Hamilton BE, Martin JA, Osterman MJ, Curtin SC, Matthews TJ (2015). Births: Final Data for 2014. National vital statistics reports: from the Centers for Disease Control and Prevention, National Center for Health Statistics, National Vital Statistics System.

[40] Aviv A, Anderson JJ, Shay JW (2017). Mutations, Cancer and the Telomere Length Paradox. Trends in cancer.

[41] Steenstrup T (2017). Telomeres and the natural lifespan limit in humans. Aging.

[42] Eisenberg DT, Tackney J, Cawthon RM, Cloutier CT, Hawkes K (2017). Paternal and grandpaternal ages at conception and descendant telomere lengths in chimpanzees and humans. American journal of physical anthropology.

[43] Eisenberg, D. T. A. & Kuzawa, C. W. The paternal age at conception effect on offspring telomere length: mechanistic, comparative and adaptive perspectives. *Philosophical transactions of the Royal Society of London. Series B, Biological sciences* **373**, 10.1098/rstb.2016.0442 (2018). 10.1098/rstb.2016.0442 PMC5784062 29335366

[44] Aviv, A. The mitochondrial genome, paternal age and telomere length in humans. *Philosophical transactions of the Royal Society of London. Series B, Biological sciences* **373**, 10.1098/rstb.2017.0210 (2018). 10.1098/rstb.2017.0210 PMC5784075 29335382

[45] Bray I, Gunnell D, Davey Smith G (2006). Advanced paternal age: how old is too old?. Journal of epidemiology and community health.

[46] Martin JA (2011). Births: final data for 2009. National vital statistics reports: from the Centers for Disease Control and Prevention, National Center for Health Statistics, National Vital Statistics System.

[47] Office for National Statistics. Birth Statistics: Birth Patterns of family building in England and Wales (FM1) (2002).

[48] Riccardi VM (1988). American paternal age data for selected years from 1876 to 1981. Neurofibromatosis.

[49] Tuljapurkar SD, Puleston CO, Gurven MD (2007). Why men matter: mating patterns drive evolution of human lifespan. PloS one.

[50] Cigno A, Ermisch J (1989). A microeconomic analysis of the timing of births. European Economic Review.

[51] Gustafsson S (2001). Optimal Age at Motherhood. Theoretical and Empirical Considerations on Postponement of Maternity in Europe. Journal of Population Economics.

[52] Klevmarken NA (2004). On the wealth dynamics of Swedish Families, 1984-98. The Review of Income and Wealth.

[53] Wilson M, Daly M (1997). Life expectancy, economic inequality, homicide, and reproductive timing in Chicago neighbourhoods. BMJ (Clinical research ed.).

[54] Waynforth D (2012). Life-history theory, chronic childhood illness and the timing of first reproduction in a British birth cohort. Proceedings. Biological sciences.

[55] Zajacova A, Dowd JB, Schoeni RF, Wallace RB (2015). Employment and income losses among cancer survivors: Estimates from a national longitudinal survey of American families. Cancer.

[56] Wozniak K, Izycki D (2014). Cancer: a family at risk. Przeglad menopauzalny = Menopause review.

