# Supplementary material for: Paternal Age and Transgenerational Telomere Length Maintenance: A Simulation Model
Source: Sci Rep. 2019 Jan 10;9:20. doi: 10.1038/s41598-018-36923-x (PMC6328556; doi:10.1038/s41598-018-36923-x)
Supplement: Supplementary file 1 — Supplementary Information [file 41598_2018_36923_MOESM1_ESM.docx]

Paternal Age and Transgenerational Telomere Length Maintenance: A Simulation Model

**Authors**: K. Horvath,^1^ D. Eisenberg,^2^ R. Stone,^1^ J. Anderson, ^3^ J. Kark,^4^ A. Aviv^1^*

**Affiliations**

^1^Center of Human Development and Aging, Rutgers, The State University of New Jersey,

New Jersey Medical School, Newark, New Jersey, United States of America

^2^Department of Anthropology, and Center for Studies in Demography and Ecology,

University of Washington, Seattle, Washington, United States of America

^3^University of Washington, School of Aquatic and Fishery Sciences, Seattle, Washington,

United States of America

^4^ Hebrew University-Hadassah School of Public Health and Community Medicine, Jerusalem, Israel

**Corresponding Author**

*avivab@njms.rutgers.edu

**Short Title**

Paternal Age and Telomere Length

**Supplementary Information**

**Table S1.** **CDC Mortality and Pregnancy Rates.** Incidence is per 100,000 individuals per year. Mortality rates include all-cause mortality adjusted to exclude malignant neoplasms (i.e. cancer).


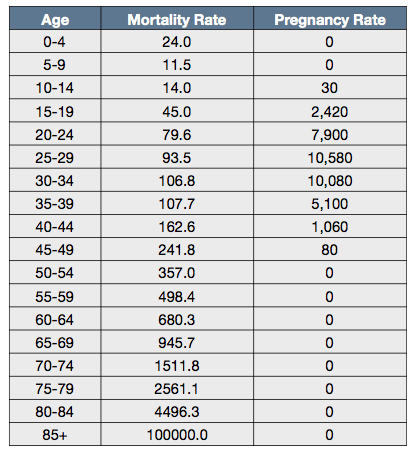


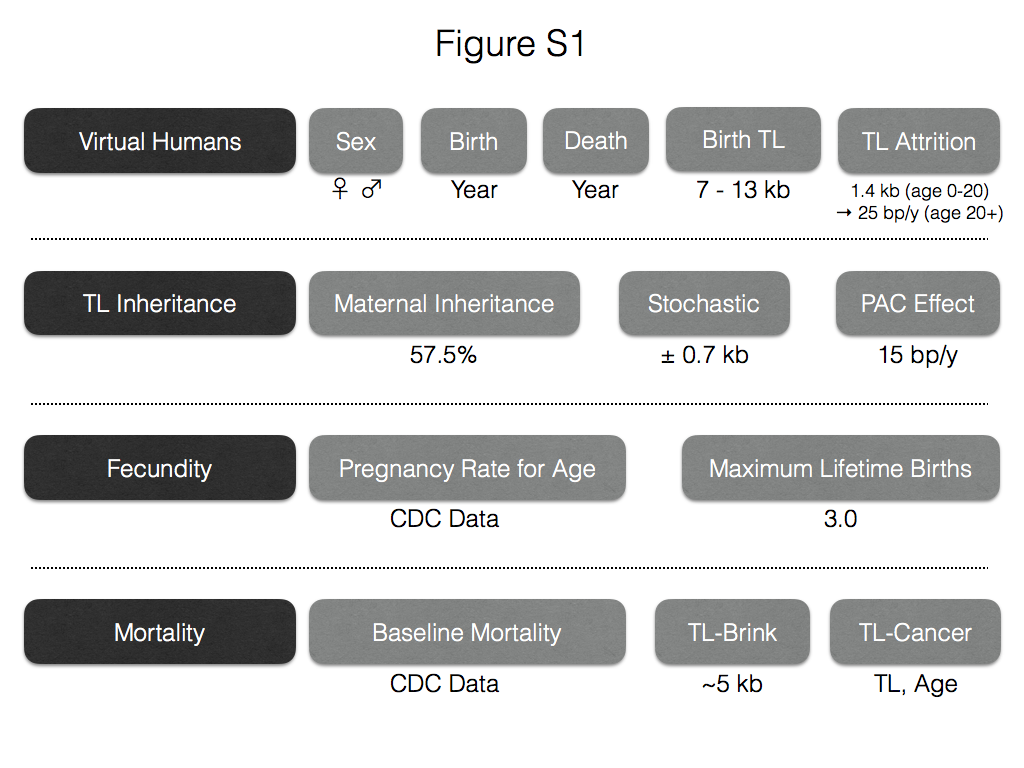
Figure S1

**Fig. S1. Summary of the four modules used in the simulations.** Simulation parameters are divided into four modules: (1) a Virtual Human module is parameterized by sex (male or female), birth year, death year, birth TL (ranging from 7 - 13 kb for the founding population), and rate of TL attrition taken as 1.4 kb in the first 20 years of life and 25 bp/year thereafter; (2) a TL Inheritance module specifies the sex-specific weighted inheritance factors (weighted at 57.5% for maternal inheritance and 42.5% for paternal inheritance), a stochastic element drawn from a normal distribution with a mean of 0 bp and standard deviation of 700 bp, and a PAC effect that contributes 15 bp /year; (3) a Fecundity module specifies the probability with which females become pregnant for a given age (taken from CDC data), and further restricts the maximum number of lifetime births to 3 children for an individual; (4) a Mortality module specifies the baseline mortality rate for a population (taken from CDC data), as well as 'telomeric brink' mortality rate indicating the probability with which an individual will die from short TL in a given year (with 50% probability of death occurring when TL is 5 kb), and a 'telomeric cancer' morality rate indicating the probability that an individual will die from cancer for at a given age for a given telomere length.

Figure S2

**Fig. S2.** **Simulation and Model Flow Chart.** Initialize Panel: The simulation begins with a population of 1,000 individuals with telomere length (TL), ranging between 5,000 to 15,000 bp. Simulation Loop Panel: The simulation then proceeds iteratively in discrete units of 1 year. Each year, all pregnant females are randomly paired with a living fertile male from the population for the purposes of determining TL in the resulting child. Each offspring is randomly assigned a sex. Birth Rate Panel: Pregnancy years for females are predetermined at birth and are determined by a stochastic process according to CDC pregnancy rates and with the constraint that no pregnancies occur in two consecutive years. At most three pregnancy- years combination of three independent processes as described in the Materials and Methods. Death Rate Panel: The death year of the individual is determined by three independent mortality processes: a baseline mortality rate drawn from CDC all-cause mortality data that is adjusted to exclude malignant neoplasms (i.e. cancer), a telomeric brink (TB) mortality process that is dependent on an individual's TL in a given year, and a cancer mortality process that is dependent on an individual's TL and age in a given year. To account for increased incidence of cancer, a constant scaling factor multiplies the cancer risk for each model. These three processes give rise to one or more possible causes of death and the earliest year is taken as the death year for the individual.

Figure S3


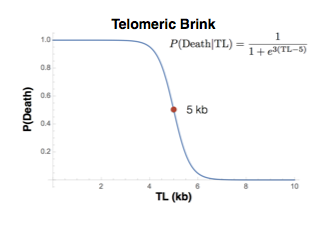


**Fig. S3.** **The telomeric brink (TB).** The TB is modelled as a probability of death for a particular year, conditioned on telomere length (TL).

Figure S4


**Fig. S4.** **The paternal-age-at-conception (PAC) effect.** The PAC mode lines depict effect of PAC on offspring telomere length (TL). Fertility is confined within ages 15 to 55 years. The effect (∆) on TL in the next generation is positive (lengthening) or negative (shortening) depending on the PAC mode. The three modes are: positive effect (PAC**^+^**), negative effect (PAC^–^) and bidirectional effect (PAC**^±^**). The isometric PAC-midpoints (PAC_mp_) denotes no change in TL in the next generation.
